# Longer Photoperiod Substantially Increases Indoor-Grown Cannabis’ Yield and Quality: A Study of Two High-THC Cultivars Grown under 12 h vs. 13 h Days

**DOI:** 10.3390/plants13030433

**Published:** 2024-02-01

**Authors:** Ashleigh Ahrens, David Llewellyn, Youbin Zheng

**Affiliations:** School of Environmental Sciences, University of Guelph, Guelph, ON N1G 2W1, Canada; aahrens@uoguelph.ca (A.A.); dllewell@uoguelph.ca (D.L.)

**Keywords:** daylength, medicinal cannabis, lighting, controlled environment agriculture, flower initiation

## Abstract

Indoor-grown *Cannabis sativa* is commonly transitioned to a 12 h daily photoperiod to promote flowering. However, our previous research has shown that some indoor-grown cannabis cultivars can initiate strong flowering responses under daily photoperiods longer than 12 h. Since longer photoperiods inherently provide higher daily light integrals (DLIs), they may also increase growth and yield. To test this hypothesis, two THC-dominant cannabis cultivars, ‘Incredible Milk’ (IM) and ‘Gorilla Glue’ (GG), were grown to commercial maturity at a canopy level PPFD of 540 µmol·m^−2^·s^−1^ from white LEDS under 12 h or 13 h daily photoperiods, resulting in DLIs of 23.8 and 25.7 mol·m^−2^·d^−1^, respectively. Both treatments were harvested when the plants in the 12 h treatment reached maturity according to established commercial protocols. There was no delay in flowering initiation time in GG, but flowering initiation in IM was delayed by about 1.5 d under 13 h. Stigma browning and trichome ambering also occurred earlier and progressed faster in the 12 h treatment in both cultivars. The vegetative growth of IM plants in the 13 h treatment was greater and more robust. The inflorescence yields were strikingly higher in the 13 h vs. 12 h treatment, i.e., 1.35 times and 1.50 times higher in IM and GG, respectively, which is 4 to 6 times higher than the relative increase in DLIs. The inflorescence concentrations of major cannabinoids in the 13 h treatment were either higher or not different from the 12 h treatment in both cultivars. These results suggest that there may be substantial commercial benefits for using photoperiods longer than 12 h for increasing inflorescence yields without decreasing cannabinoid concentrations in some cannabis cultivars grown in indoor environments.

## 1. Introduction

*Cannabis sativa* (hereafter, cannabis) is a dioecious annual that has been domesticated worldwide for medicinal, textile, recreational, and nutritional uses. Cannabis is well known for the cannabinoids it produces such as tetrahydrocannabinol (THC) and cannabidiol (CBD), which are produced in high abundance in stalked glandular trichomes associated with unfertilized floral tissues of female plants (Small, 2016) [1].

Most cannabis cultivars that are grown commercially in indoor environments are drug-type (i.e., THC content > 0.3%), due to the relatively high value of their mature female floral tissues. Indoor-grown cannabis cultivars typically have short-day photoperiod responses, whereby daylengths greater than 16 h (i.e., uninterrupted dark periods ≤ 8 h) will keep plants in the vegetative growth stage while shortening the photoperiod below some critical level will transition plants toward producing generative tissues (i.e., flowering initiation).

The robustness of photoperiodic cannabis’ flowering response tends to increase as the daylength continues to be reduced below the critical photoperiod. Many modern drug-type cannabis cultivars have been developed by hybridizing cannabis cultivars grown from a broad range of latitudes with different seasonal photoperiod dynamics (Small, 2022) [2]. Therefore, modern cannabis cultivars may have considerable variability in their optimum daylengths for promoting robust flowering responses and maximizing yield and quality. The range of photoperiodic responses has been well documented in hemp cultivars (e.g., Zhang et al., 2021) [3], but the studies by Peterswald et al. (2023) [4] and Ahrens et al. (2023) [5] are the only recent studies on photoperiodic responses of modern indoor-grown cannabis cultivars. Indoor-grown cannabis crops are almost universally switched to a 12-h daily photoperiod to initiate the flowering stage of production (Potter, 2014) [6]. This optimizes operational simplicity in cultivation systems that grow many different cultivars concurrently. However, recent studies have shown that some modern indoor-grown cannabis cultivars have robust flowering responses to moderately longer photoperiods (Peterswald et al., 2023; Ahrens et al., 2023; Potter, 2009) [4,5,7]. Longer photoperiods have the advantages of either (1) increased daily light integrals (DLIs), which may result in higher yields (Rodriguez-Morrison et al., 2020) [8], or (2) lower installed lighting levels (e.g., reduced fixture density), which reduces upfront lighting infrastructure and installation costs.

Our recent study demonstrated that about half of the ten investigated cultivars had minimal delays in flowering initiation in photoperiods up to 13.5 h (Ahrens et al., 2023) [5]. However, this study ended well before the cultivars reached normal commercial maturity. Therefore, the effects of longer photoperiods on mature inflorescence yield and quality are still unknown. The objective of the present study was to compare the yield and quality of two cultivars (selected from the study by Ahrens et al. (2023) [5]) grown to commercial maturity under 12 h or 13 h photoperiods. The hypotheses were that inflorescence yield would be proportional to the DLI associated with each photoperiod and that there would be no photoperiod treatment effects on the concentrations of the major cannabinoids.

## 2. Materials and Methods

### 2.1. Experimental Design and Plant Cultivation

This trial was conducted in a walk-in growth chamber at the University of Guelph with two rows of three compartments separated by opaque white curtains to prevent inter-compartment light contamination, as described in the study by Ahrens et al. (2023) [5]. Two full spectrum LED fixtures (Jungle—LED G4i 1200, Allstate Garden Supply, Ontario, CA, USA) were hung in each compartment on height-adjustable pulley systems. These fixtures have onboard dimmers with fixed settings of 25%, 50%, 75%, and 100% of maximum intensity. The relative spectral photon flux distribution of the LED fixtures is provided in Figure S1, which was the same as in the study by Ahrens et al. (2023) [5].

Two cannabis cultivars, ‘Incredible Milk’ (IM) and ‘Gorilla Glue’ (GG), were sourced from a single commercial cultivator in Southwestern Ontario. Seventy-eight stem tip cuttings were taken from vegetative mother plants of each cultivar and inserted into 3.6 × 4.0 cm round rockwool plugs (Macroplug; Grodan, Milton, ON, Canada) on 7 November 2022 at the cultivator’s facility. The rooted cuttings were delivered to the University of Guelph on 29 November 2022 and transplanted on 1 December 2022 into presoaked 10 × 10 × 7.5 cm rockwool blocks (Grodan GRO-BLOCK Improved GR7.5 Medium 4”; Grodan). Transplants were grown vegetatively (18 h light/6 h dark) with the LEDs at 85 cm above the canopy. The dimmers were set at 25% on 29 November 2022, which provided an average canopy-level photosynthetic photon flux density (PPFD) of ≈135 µmol·m^−2^·s^−1^. Dimmer settings were incrementally increased every two days until reaching 100% (≈540 µmol·m^−2^·s^−1^) on 5 December 2022.

Eighteen uniformly sized plants of each cultivar were randomly assigned to one of two photoperiod treatments on 5 December 2022: 12 h light/12 h dark (12 h) or 13 h light/11 h dark (13 h). All LED fixtures were set to maximum intensity, resulting in uniform PPFD of ≈540 µmol·m^−2^·s^−1^ at an 85 cm hang height. The daily light integrals for the 12 h and 13 h photoperiods were 23.8 and 25.7 mol·m^−2^·d^−1^, respectively. The photoperiod treatments were randomly assigned to individual compartments, resulting in 3 concurrent replications of each treatment. Three plants of each cultivar were placed onto presoaked 100 × 15 × 7.5 cm rockwool slabs (Vital; Grodan), one slab per cultivar, in each compartment. The locations of each cultivar’s slab were randomized in each compartment (Figure S2). Each slab was centered in a subirrigation tray (53 × 109 cm grow trays, Botanicare, Vancouver, WA, USA). The plants were 35 cm apart on the slab and 70 cm apart between slabs measured ‘on center’ (Figure S3). The plants were subirrigated as needed for the first 9 d after the photoperiod treatments began and then drip-irrigated henceforth. One irrigation dripper (Supertif PCND-MOP 1.1 L·h^−1^; Rivulis, Gvat, Israel) was placed in each rockwool block, and two drippers were placed in each rockwool slab, centered between adjacent plants (Figure S3). There were eight irrigation events per day, with event lengths increased, as needed, to maintain a daily leachate percentage of at least 15%. The first irrigation event occurred ≤ 1 h after lights turned on, followed by 1 h rest periods between the next five irrigations and 2 h rest periods between the last two irrigations. The length of individual irrigations was the same throughout any given day but increased from 120 s at the beginning of the trial to 420 s by the end, to maintain ≥ 15% daily leachate levels. The cannabis flowering nutrient recipe from Zheng (2022) [9] was used, mixed in deionized water to an electrical conductivity of 2.0 mS·cm^−1^, and pH was adjusted to 5.7 with a 1.0 M potassium bicarbonate (Master Plant-Prod Inc., Brampton, ON, Canada).

The chamber temperature was set at a constant 25 °C, and relative humidity (RH) was set at 70% light/60% dark, which was controlled with a fogging system connected to the climate computer (Titan I/O Plus, Argus Control Systems Limited, Surrey, BC, Canada). The LEDs were turned on daily at 09:00 and turned off at 21:00 and 22:00 for the 12-h and 13-h treatments, respectively. The photoperiod was controlled with the computer. The curtains at the front of each compartment were opened daily at 09:00 and closed daily at 21:00. The LED fixtures were raised weekly to maintain the 85 cm distance between the canopy and the LEDs. There was no CO_2_ supplementation in this trial, but ample air exchanges ensured CO_2_ concentrations were consistently >400 PPM during lit periods.

### 2.2. Data Collection

Temperature and relative humidity data were logged every 300 s using three dataloggers (MX2301A; HOBO; Onset Computer Corporation; Bourne, MA, USA), with each logger assigned to one replicate compartment from each treatment. The loggers were switched between their respective compartments daily. The day and night aerial temperature and RH for each compartment are summarized in Table S1.

Elapsed days to flowering (EDTF), which is defined as the number of days between invoking the photoperiod treatments and the appearance of ≥3 pairs of stigmas (i.e., an inflorescence) at the top of the primary shoot, was monitored daily starting at 1 d after the photoperiod treatments began. The apical inflorescence was defined as the conglomeration of inflorescence and foliar tissues (i.e., sugar leaves) with no visible gaps located at the top of the primary shoot. The percentage of brown stigmas and amber trichomes on the apical inflorescence were monitored daily to track inflorescence maturity, beginning 37 d after photoperiod treatments began.

The harvesting of each cultivar began after >95% of the stigmas in either photoperiod treatment had turned brown, and either 10% of the trichomes had turned to an amber color (IM) or the fan leaves began senescing (GG), whichever occurred first. The harvesting of IM and GG began on days 58 and 72, respectively, after initiating the photoperiod treatments. One replicate of each treatment (i.e., six plants) was harvested per day, for three successive days.

The harvest process followed the study by Ahrens et al. (2023) [5] with the addition of separating the vegetative tissues into fan leaves, sugar leaves, and stems. To differentiate between fan and sugar leaves, any leaf with ≥1 cm of petiole visible when the longitudinal axis of the plant was at the nadir with respect to the observer (i.e., looking straight downwards from the top of the plant) was considered a fan leaf. Prior to cutting the plant at the substrate surface (i.e., the top of the rockwool blocks), plant height and width (at the widest point and perpendicular to this) were measured to determine the growth index following the study by Ruter (1992) [10]. The apical inflorescences were separated from the rest of the plants to determine their volume (following Ahrens et al., 2023 [5]) and fresh weight (FW). The apical inflorescence FW was divided by volume to determine apical inflorescence density (g·cm^−3^). The apical inflorescence of the most representative plant from each photoperiod by cultivar combination in each replicate was air-dried at 23 °C and 70% RH to approximately 10% moisture content. During the harvesting process, photographs were taken of the whole plant, the whole plant with fan leaves removed, and the excised apical inflorescence of the most representative plant in each photoperiod by cultivar combination in each replicate. The FW of the fan leaves, sugar leaves, stems, and the nonapical inflorescence tissues of each plant were recorded. The harvest index was calculated as follows: total inflorescence FW/(total inflorescence FW + aboveground vegetative FW). The separated tissues of each photographed plant were dried to constant weight at 70 °C. The representative plants’ tissue dry weights (DWs) were then used to calculate the moisture content of each tissue type, which were then used to estimate the DWs of the remaining plants in each respective cultivar by photoperiod combination. All tissue weights were measured in grams using a digital balance (BCE2202-1S; Sartorius Lab Instruments, Göttingen, Germany).

### 2.3. Cannabinoid Analysis

The air-dried apical inflorescences of all of the representative plants were sent to a third-party lab (A&L Canada Laboratories Inc., London, ON, Canada) for the analysis of moisture content (i.e., loss on drying) and the concentrations of the following cannabinoids: cannabigerolic acid (CBGA), cannabigerol (CBG), cannabidiolic acid (CBDA), cannabidiol (CBD), cannabinolic acid (CBNA), cannabinol (CBN), cannabidivarinic acid (CBDVA), cannabidivarin (CBDV), Δ^9^-tetrahydrocannabinolic acid (THCA), Δ^9^-tetrahydrocannabinol (THC), tetrahydrocannabivarinic acid (THCVA), and tetrahydrocannabivarin (THCV). The total equivalent THC (T-THC) is an estimation of the amount of THC available to the consumer, once THCA has been decarboxylated into THC, such as from adding heat. The equation used is T-THC = (THCA × 0.877) + THC. The limit of quantitation (i.e., LOQ) for these analyses was reported as <0.05% for each cannabinoid. All cannabinoid concentrations are reported as the percentage of dry weight, based on the loss-on-drying determination of the sampled tissues’ moisture content. The total inflorescence dry weight was calculated by adding nonapical inflorescence dry weight (oven-dried) and apical inflorescence dry weight (loss on drying) for each plant.

### 2.4. Statistical Analysis

The experiment was a completely randomized design, with two treatments and three replications. Statistical analysis was performed using RStudio (v2021.9.0.351; Posit Software, PBC; Boston, MA, USA). The parameter means of each replicate in each cultivar by treatment combination were analyzed for the EDTF, harvest, and postharvest measurements (*n* = 3). Each sample was considered a replicate in the cannabinoid data (*n* = 3). Unpaired, two-tailed Student’s *t*-tests were performed between the 12 h and 13 h treatments for each parameter by cultivar combination. Statistical significance was determined at the *p* ≤ 0.05 level. 

## 3. Results

### 3.1. Elapsed Days to Flowering (EDTF)

**Figure 1:**
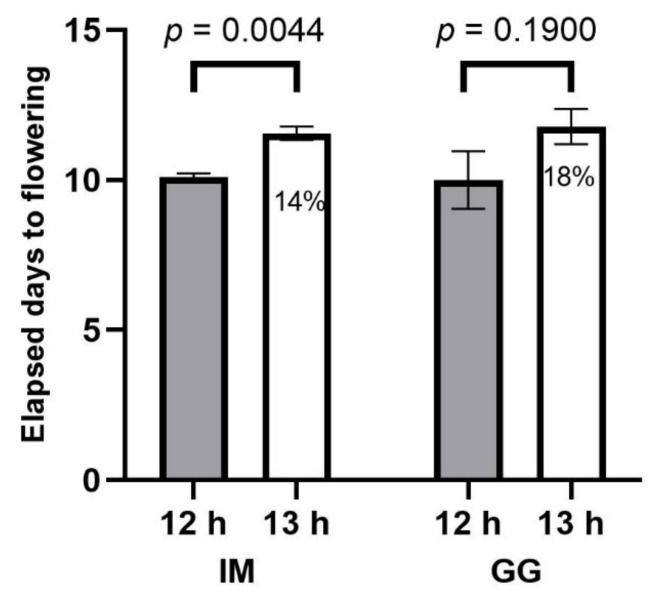
Elapsed days to flowering (EDTF) responses to 12 h (filled bars) and 13 h (empty bars) photoperiod treatments of *C. sativa* cultivars ‘Incredible Milk’ (IM) and ‘Gorilla Glue’ (GG). Data are means ± SE, *n* = 3. Error bars are presented for all data but may be obscured for small SE values. Percentage values marked in the 13 h bars signify the increase in EDTF relative to the 12 h treatment, and the *p*-values above each cultivar represent the significance level of the comparison of means according to Student’s *t*-test.

**Figure 2:**
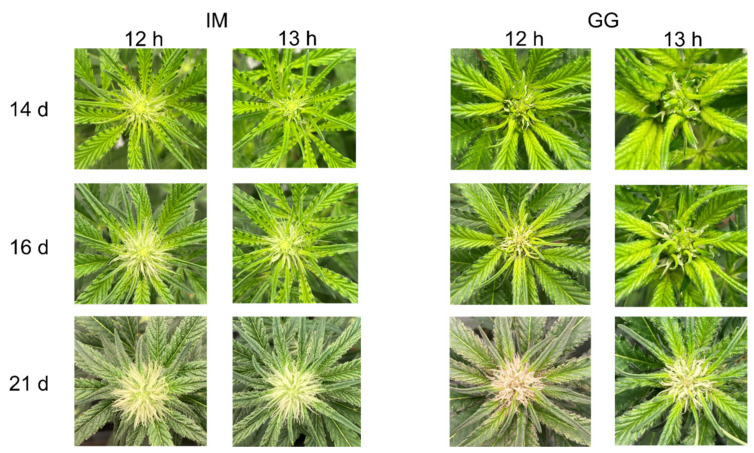
Representative photos showing early apical inflorescence development of *C. sativa* cultivars ‘Incredible Milk’ (IM) and ‘Gorilla Glue’ (GG) under 12 h and 13 h photoperiod treatments from 14 d to 21 d after the start of photoperiod treatments.

**Figure 3:**
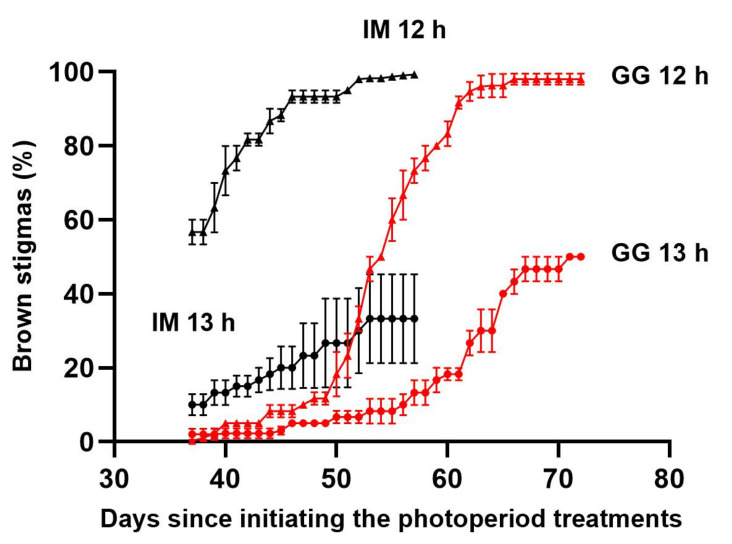
Temporal dynamics of stigma browning on the apical inflorescence of the primary shoot of *C. sativa* cultivars ‘Incredible Milk’ (IM, black) and ‘Gorilla Glue’ (GG, red) grown under 12 h (triangles) and 13 h (circles) photoperiod treatments. Data are means ± SE of three replicates (*n* = 3). Error bars are presented for all data but may be obscured for small SE values.

The initiation of flowering of IM was delayed in the 13 h treatment by approximately 1.5 d, but there were no photoperiod treatment effects on EDTF in GG (Figure 1). However, the rate of early inflorescence development appeared to be slightly delayed in the 13 h treatment in both cultivars (Figure 2). Stigma browning was substantially delayed in the 13 h treatment in both cultivars (Figure 3).

### 3.2. Inflorescence Yield

**Figure 4:**
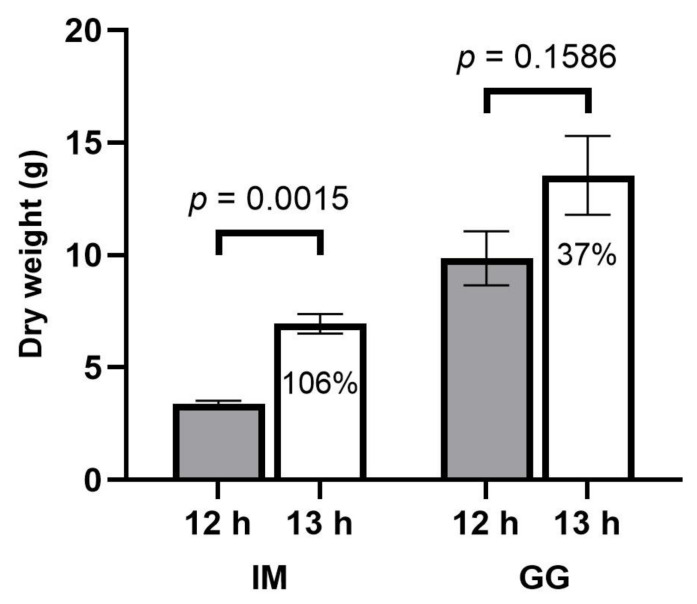
Dry weight responses of individual plants’ apical inflorescence to 12 h (filled bars) and 13 h (empty bars) photoperiod treatments of *C. sativa* cultivars ‘Incredible Milk’ (IM) and ‘Gorilla Glue’ (GG). Data are means ± SE, *n* = 3. Error bars are presented for all data but may be obscured for small SE values. Percentage values marked in the 13 h bars signify the increase in apical inflorescence dry weight relative to the 12 h treatment, and the *p*-values above each cultivar represent the significance level of the comparison of means according to Student’s *t*-test.

**Figure 5:**
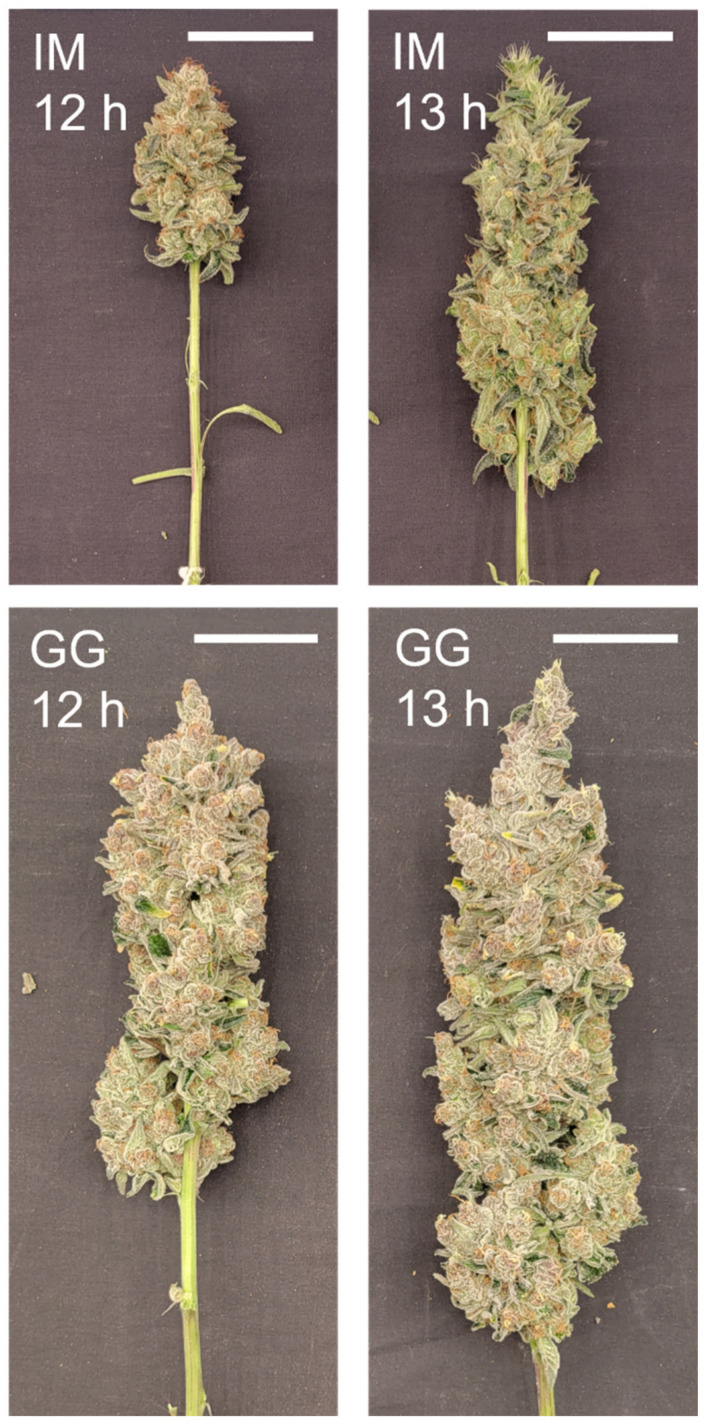
Images of the apical inflorescence of the primary shoot of representative plants at harvest of *C. sativa* ‘Incredible Milk’ (IM) plants on day 58 and of ‘Gorilla Glue’ (GG) on day 72 after the start of the photoperiod treatments. The white scale bars in each image are 5 cm.

There were no photoperiod treatment effects on apical inflorescence DW in GG, but the apical inflorescence DW in IM more than doubled under the 13 h vs. 12 h treatments (Figure 4 and Figure 5).

**Figure 6:**
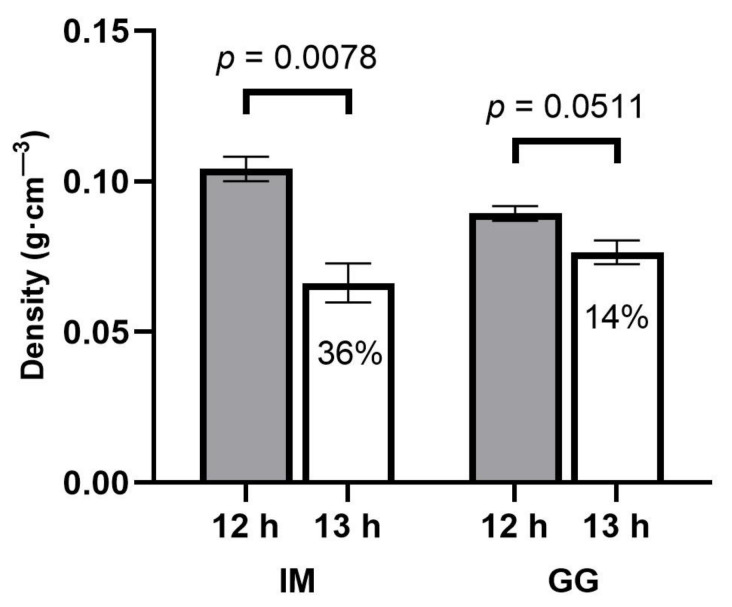
Apical inflorescence density responses to 12 h (filled bars) and 13 h (empty bars) photoperiod treatments of *C. sativa* cultivars ‘Incredible Milk’ (IM) and ‘Gorilla Glue’ (GG). Data are means ± SE, *n* = 3. Error bars are presented for all data but may be obscured for small SE values. Percentage values marked in the 13 h bars signify the decrease in apical inflorescence density relative to the 12 h treatment, and the *p*-values above each cultivar represent the significance level of the comparison of means according to Student’s *t*-test.

There were no photoperiod treatment effects on apical inflorescence density in GG, but the apical inflorescence density of IM was 36% lower in the 13 h treatment (Figure 6).

**Figure 7:**
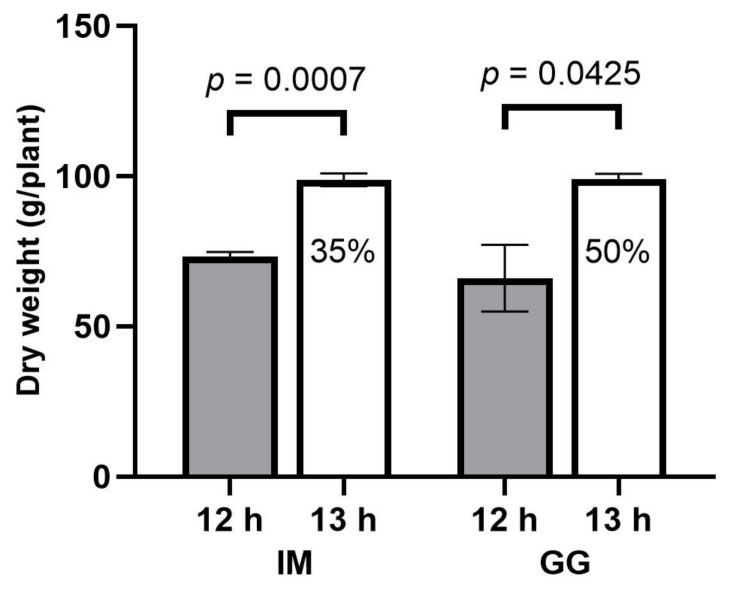
Total inflorescence dry weight responses (g/plant) to 12 h (filled bars) and 13 h (empty bars) photoperiod treatments of *C. sativa* cultivars ‘Incredible Milk’ (IM) and ‘Gorilla Glue’ (GG). Total inflorescence dry weight was calculated by adding nonapical inflorescence dry weight (oven-dried) and apical inflorescence dry weight for each plant. Data are means ± SE, *n* = 3. Error bars are presented for all data but may be obscured for small SE values. Percentage values marked in the 13 h bars signify the increase in total inflorescence dry weight relative to the 12 h treatment, and the *p*-values above each cultivar represent the significance level of the comparison of means according to Student’s *t*-test.

The total inflorescence DW was 35% higher in IM and 50% higher in GG in the 13 h vs. 12 h treatments (Figure 7).

### 3.3. Apical Inflorescence Cannabinoid Concentrations

**Figure 8:**
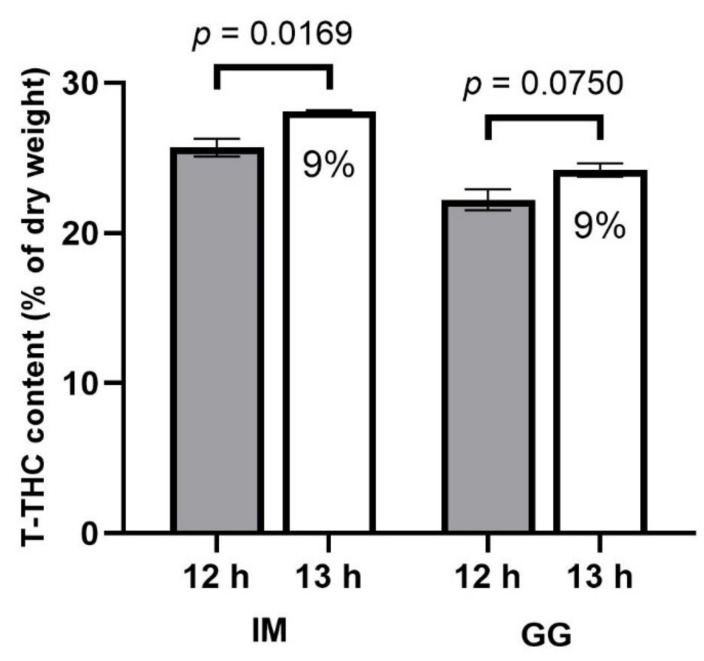
Total equivalent THC (T-THC) concentrations (% of dry weight) in the apical inflorescence responses to 12 h (filled bars) and 13 h (empty bars) photoperiod treatments of *C. sativa* cultivars, ‘Incredible Milk’ (IM) and ‘Gorilla Glue’ (GG). Data are based on the loss-on-drying determinations of the water content of the sampled tissues. Data are means ± SE, *n* = 3. Error bars are presented for all data but may be obscured for small SE values. Percentage values marked in the 13 h bars signify the increase in T-THC relative to the 12 h treatment, and the *p*-values above each cultivar represent the significance level of the comparison of means according to Student’s *t*-test.

Both cultivars in this study had relatively low CBD and high THC concentrations (Table 1). The CBGA and CBDA concentrations were 53% and 19% higher, respectively, in the 13 h treatment in IM. There were no photoperiod treatment effects on CBG concentration in either cultivar. The THCA concentration was 10% higher in the 13 h treatment in IM. There were no photoperiod treatment effects in Δ^9^-THC concentration in either cultivar. The T-THC concentration in IM was 9% higher in the 13 h treatment, but there were no photoperiod treatment effects on T-THC content in GG (Figure 8). The THCVA concentrations were 22% and 16% higher in the 13 h vs. 12 h photoperiod treatments in IM and GG, respectively. All other measured cannabinoids were below the limit of quantitation (i.e., <0.05%).

### 3.4. Vegetative Growth, Weight of Aboveground Tissues, and Harvest Index

**Figure 9:**
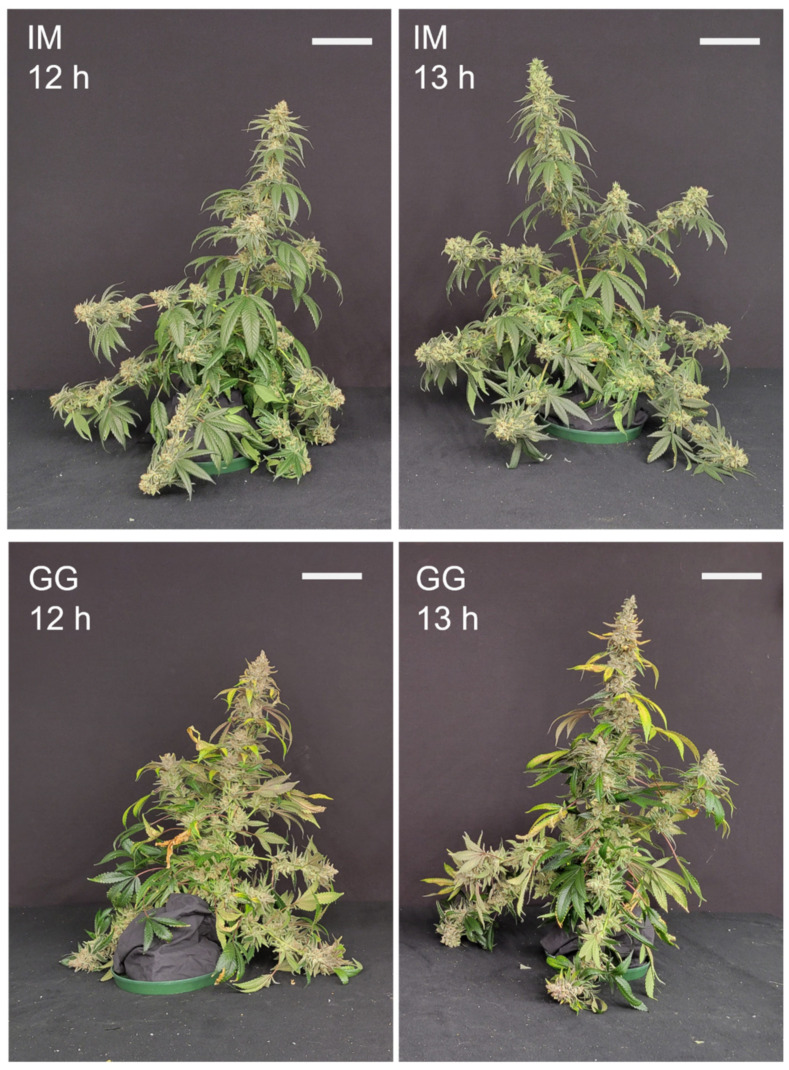
Whole-plant images taken just before the harvest of representative plants of *C. sativa* ‘Incredible Milk’ (IM) and ‘Gorilla Glue’ (GG) on days 58 and 72, respectively, after the start of the photoperiod treatments. The white scale bars in each image are 15 cm.

**Figure 10:**
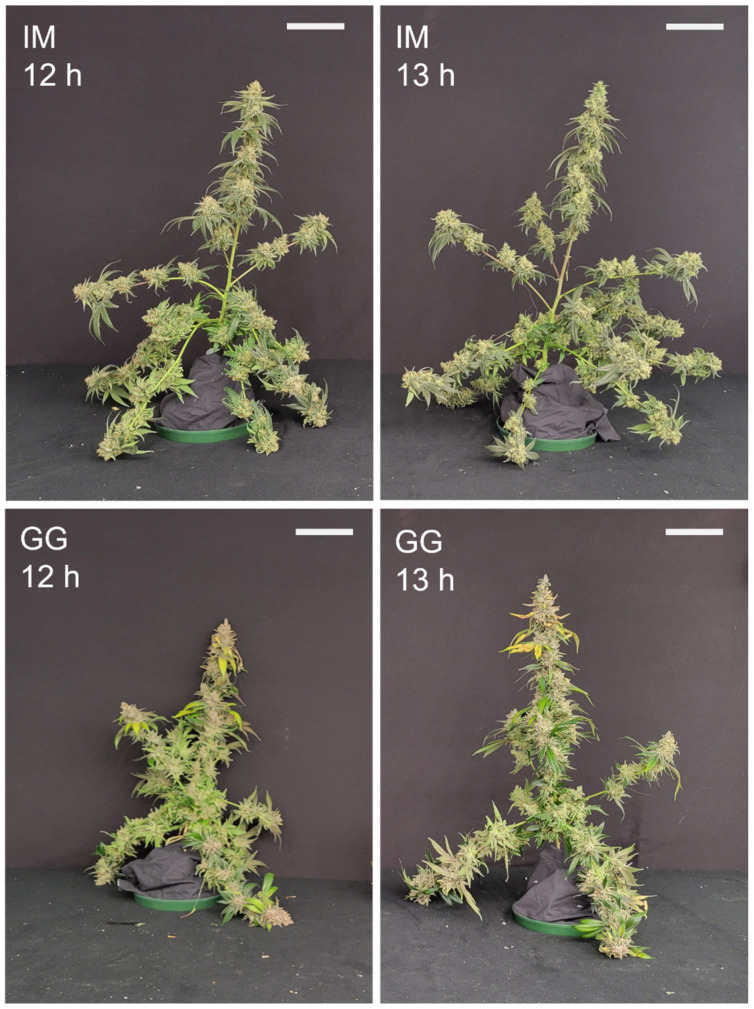
Whole-plant images with the fan leaves removed of representative plants of *C. sativa* ‘Incredible Milk’ (IM) and ‘Gorilla Glue’ (GG) on days 58 and 72, respectively, after the start of the photoperiod treatments. The white scale bars in each image are 15 cm.

**Figure 11:**
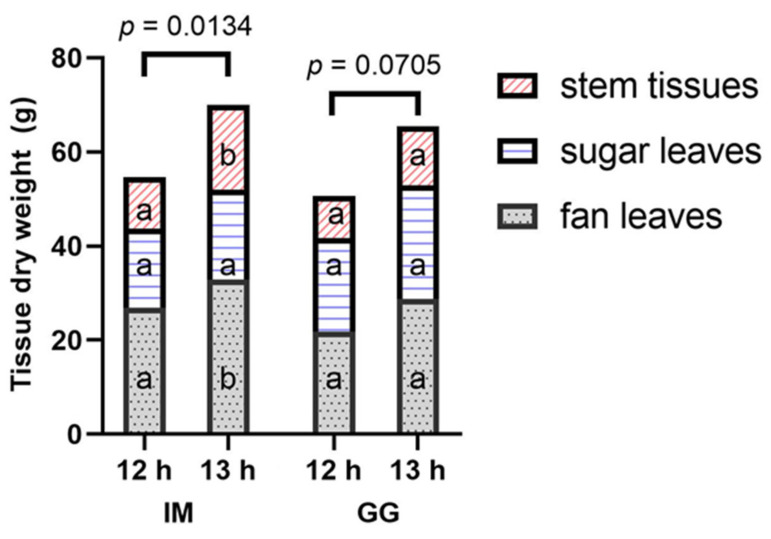
Dry weight (mean, *n* = 3) of aboveground vegetative tissues from individual plants under 12 h and 13 h photoperiod treatments in *C. sativa* cultivars ‘Incredible Milk’ (IM) and ‘Gorilla Glue’ (GG). Tissues are separated into stems (diagonal pink lines), sugar leaves (horizontal blue lines), and fan leaves (gray dotted pattern). In each cultivar and each tissue type, photoperiod treatments bearing the same lowercase letter are not significantly different at *p* ≤ 0.05 according to Student’s *t*-test. The *p*-values above each cultivar represent the significance level of the comparison of the total aboveground vegetative DW according to Student’s *t*-test.

The plants of both cultivars appeared larger in the 13 h vs. 12 h photoperiod (Figure 9). With the fan leaves removed, the plants of both cultivars in the 13 h treatment appeared to have more robust branches with stronger support for the inflorescence tissues (Figure 10), despite also bearing the weight of substantially more inflorescence biomass (Figure 6). The dry weights (DWs) of fan leaves, stem tissues (including side branches), and the total aboveground vegetative biomass were 22%, 65%, and 28% higher in the 13 h treatment in IM (Figure 11). There were no photoperiod treatment effects on the DWs of any aboveground vegetative tissues in GG. The growth index of IM was 81% higher in the 13 h treatment (Table 2). The harvest index of GG was 6% higher in the 13 h treatment. The total dry weights of aboveground tissues (i.e., including inflorescence tissues) were 32% higher in IM and 41% in GG in the 13 h treatment.

## 4. Discussion

An almost universal practice in indoor cannabis cultivation is to reduce the daily photoperiod to 12 h to transition vegetative plants to flowering. While this protocol works for virtually all indoor-grown cannabis cultivars, some cultivars have robust flowering responses to photoperiods that are modestly longer than 12 h (Peterswald et al., 2023; Ahrens et al., 2023) [4,5]. Since higher light levels can increase inflorescence yield and quality (Rodriguez-Morrison et al., 2021; Llewellyn et al., 2022) [8,11], longer flowering-stage photoperiods (with corresponding increases in DLIs) may have positive influences on cannabis inflorescence yield and quality. Also, in greenhouse production during summer months in higher latitude regions (e.g., Canada), blackout curtains can remain open longer, allowing for better utilization of natural light and reductions in some other climate-control-related costs. A 12 h flowering-stage photoperiod may not be optimized for maximizing the yield of all cultivars. Hence, cultivators who use a 12 h photoperiod for all cultivars may be ‘leaving yield on the floor’. The ≥35% increases in the total inflorescence yield in the 13 h treatment observed in the present study were similar to the yield increases in the 14 h vs. 12 h photoperiod reported by Peterswald et al. (2023) [4], who attributed the higher yields to increased DLIs. However, in both the present study and that of Peterswald et al. (2023) [4], the observed yield increases under longer photoperiods were disproportionately higher than the increases in DLIs, contrary to our hypothesis. Other developmental and morphophysiological responses to longer photoperiods may be contributing to the yield enhancements.

In outdoor environments, after the summer solstice (i.e., 21 June in the Northern Hemisphere), daily photoperiods gradually reduce a few minutes each day, with daily changes in photoperiod depending on latitude and time of year. For example, in Guelph, Ontario, Canada (43.5° N, −80.2° W), there are ≈90 d between the summer solstice (15.5 h) and 12 h days (Sept 26) (Hoffmann, 2024) [12]. For short-day plants, the seasonal reductions in daylength indicate the onset of the end of the growing season; photoperiodic cannabis cultivars respond accordingly by transitioning from vegetative to generative growth, ending in floral maturation, senescence, and plant death. In contrast to the outdoor environment, reducing from ≥16 h directly to 12 h in indoor cannabis cultivation is a relatively powerful signal that the end of the growing season (e.g., the onset of killing frost) approaches. Accordingly, regardless of their provenance, virtually all indoor-grown cannabis cultivars rapidly initiate flowering under a 12 h photoperiod, normally producing visible inflorescence tissues (groupings of ≥3 stigmas) in less than two weeks and mature inflorescences six to ten weeks later (Peterswald et al., 2023; Potter, 2014; Rodriguez-Morrison et al., 2021; Llewellyn et al., 2022; Potter and Duncombe, 2012) [4,6,8,11,13].

While some indoor-grown cannabis cultivars can flower under photoperiods longer than 12 h, increasing the photoperiod may moderate the speed and intensity of the transition to reproductive growth. This is supported in the present study by the delayed time to visible inflorescences in IM and slower early floral development in both cultivars. These patterns were also reported by Ahrens et al. (2023) [5] for some cultivars, and by Zhang et al. (2021) for essential oil cultivars [3]. However, as seen in the present study, delayed or repressed flowering initiation in the 13 h photoperiod led to enhanced rather than repressed inflorescence biomass when plants reached commercial maturity. Since much of the vegetative growth after switching to the flowering photoperiod occurs during the first few weeks after the transition to short days (Potter, 2014; Yep et al., 2020) [6,14], enhancements in vegetative growth during this period increase foliar biomass, probably enhancing light interception and thus growth potential. Enhanced vegetative growth during the early phases of the flowering stage may also increase the number of potential flowering sites and capacity for structurally supporting higher floral biomass, which has been observed in vegetative-stage cannabis (Moher et al., 2022) [15] and in flowering hemp grown in protected culture (Hall et al., 2014) [16]. The plants of both cultivars in the 13 h treatment in the present study had higher growth indexes and appeared to be larger. However, increases in the DW of individual vegetative tissues in the 13 h treatment were only observed in IM. Regardless, the higher inflorescence yields in the 13 h treatment may be a consequence of generally larger plants, which are known to increase commercially mature inflorescence yield potential (Bevan et al., 2021) [17].

Despite the early delays in inflorescence development, by the time the plants in the 12 h treatment reached commercial maturity, the total inflorescence yield and the size of the apical inflorescences were markedly higher in the 13 h treatment in both cultivars. Given that the increases in inflorescence yield were disproportionately higher than the increase in DLI, flowering photoperiod management may be one of the most efficacious cultural practices available to commercial indoor cannabis cultivators for increasing yield that is both simple and cost-effective to utilize. Furthermore, despite the plants in the 13 h treatment appearing to have delayed inflorescence maturation rates (e.g., slower stigma browning and reduced trichome ambering), the cannabinoid composition in the apical inflorescences was of comparable quality in both treatments in both cultivars. There were moderately higher CBGA concentrations in the 13 h treatment in IM, which suggests that the inflorescence tissues in this treatment may have been less mature (Aizpurua-Olaizola et al., 2016) [18]. However, there was no detectable CBN in either cultivar, and the neutral form of THC was a low proportion of total THC, suggesting that neither treatment had been harvested beyond its optimum maturity level (Aizpurua-Olaizola et al., 2016; Russo, 2007) [18,19]. The CBDA levels in IM were also higher in the 13 h treatment. However, as the THCA-to-CBDA ratios were ≥400 in both cultivars, treatment effects in CBD content in the cultivars used in this study are likely not commercially relevant, at least in most markets that favor such highly THC-dominant cultivars. This is also the case with the observed treatment effects on THC concentrations, which represented only a small fraction of the total THC (T-THC) content. More relevant are the effects of photoperiod on the T-THC content, which were either insignificant (GG) or higher (IM) in the 13 h treatment. Since the 13 h treatment increased the inflorescence DW, the 13 h treatment also increased THC yield (g/plant) accordingly, by ≥38%. These results are in contrast with those obtained by Peterswald et al. (2023) [4], who found approximately 40% reductions in the floral THC concentrations in the two THC-dominant cultivars when grown in the 14 h vs. 12 h photoperiod treatment. These results are also contrary to our hypothesis, which distinguishes the present study from many contemporary cannabis lighting studies that often showed only minor effects of light treatments on cannabinoid composition (e.g., Rodriguez-Morrison et al., 2021; Llewellyn et al., 2022; Potter and Duncombe, 2012) [8,11,13]. Further study is needed to determine the mechanisms for how photoperiod, along with other environmental inputs, affect the composition of the metabolome in indoor-grown cannabis.

Another important quality aspect of cannabis is the density of the apical inflorescences, where more dense inflorescences are normally regarded as having higher quality. Apical inflorescence density has been shown to increase linearly with light intensity (Rodriguez-Morrison et al., 2021) [8]. However, inflorescence density in IM was lower in the longer photoperiod in the current study, suggesting that the developmental ramifications of longer photoperiods on apical inflorescence tissues may override the benefits of higher DLIs. Overall, aside from lower apical inflorescence density in IM, the 13 h treatment substantially increased the apical inflorescence size, total inflorescence yield, and cannabinoid yield.

Despite having similar prescribed days to maturity in commercial production (Ahrens et al., 2023) [5], GG required ≈25% longer to reach commercial maturity in the present study, regardless of the photoperiod treatment. Factoring in the relative lengths of the flowering cycle of each cultivar, IM was ≈25% more efficient (i.e., g·d^−1^) than GG at producing floral biomass in both treatments. While further study is required to elucidate the specific mechanisms of photoperiod responses of indoor-grown cannabis cultivars, the observed cultivar differences in photoperiod responses generally illustrate the need to investigate individual cultivars’ photoperiod responses under the cultivators’ specific cultivation environment for full flowering cycles.

Due to the relatively small plot sizes in the present study, the massive per-plant yield increases may be tempered by competition for space and light in commercial indoor cannabis cultivation systems, depending on the planting density. Readers are therefore cautioned against inferring per-area yield increases (e.g., over commercially relevant production areas) from the per-plant yield increases reported in the present study. Future research should explore the effects of moderately longer photoperiods than 12 h on the inflorescence yield and quality of different indoor-grown cannabis cultivars. Particular focus should be directed toward investigating narrower discreet time differences between photoperiod treatments (e.g., ≤15 min) and the effects of planting density on the treatment effects of photoperiod on yield and quality, both on per-plant and per-area bases.

## 5. Conclusions

The 13 h photoperiod treatment increased inflorescence yield disproportionately higher than the increase in DLI in both cultivars. In addition, while the longer photoperiod somewhat delayed inflorescence development, the major cannabinoid concentrations in the apical inflorescence tissues at commercial maturity were either unchanged or enhanced. Therefore, increasing the photoperiod during the flowering stage of indoor cannabis cultivation is an easily employed cultivation protocol for enhancing indoor cannabis production. However, cannabis’ photoperiod responses are strongly cultivar-dependent; growers must investigate the effects of photoperiods with their own specific cultivars and cultivation systems.

## Figures and Tables

**Table 1:** Responses of the apical inflorescences’ cannabinoid concentrations (% of dry weight) to 12 h and 13 h photoperiod treatments for *C. sativa* cultivars ‘Incredible Milk’ (IM) and ‘Gorilla Glue’ (GG). Data are based on the loss-on-drying determination of sampled tissues’ water content. Data are means ± SE, *n* = 3. Percent change values signify the change in cannabinoid content of the 13 h treatment relative to the 12 h treatment. The *p*-values are according to Student’s *t*-test.

Cultivar	Cannabinoid ^z^	12 h	13 h	Percent Change	*p*-Value ^y^
IM	CBGA	1.34 ± 0.025	2.05 ± 0.025	53%	<0.0001
	CBG	0.14 ± 0.007	0.13 ± 0.006	−1%	0.8870
	CBDA	0.07 ± 0.001	0.08 ± 0.001	19%	<0.0001
	CBD	<LOQ ^x^	<LOQ		
	CBNA	<LOQ	<LOQ		
	CBN	<LOQ	<LOQ		
	CBDVA	<LOQ	<LOQ		
	CBDV	<LOQ	<LOQ		
	THCA	28.7 ± 0.67	31.5 ± 0.16	10%	0.0152
	THC	0.51 ± 0.020	0.45 ± 0.065	−13%	0.3886
	THCVA	0.15 ± 0.007	0.18 ± 0.007	22%	0.0325
	THCV	<LOQ	<LOQ		
GG	CBGA	0.90 ± 0.059	1.13 ± 0.059	25%	0.0539
	CBG	0.12 ± 0.006	0.13 ± 0.006	11%	0.1998
	CBDA	0.05 ± 0.001 *	0.06 ± 0.004	15%	0.1790
	CBD	<LOQ	<LOQ		
	CBNA	<LOQ	<LOQ		
	CBN	<LOQ	<LOQ		
	CBDVA	<LOQ	<LOQ		
	CBDV	<LOQ	<LOQ		
	THCA	25.1 ± 0.77	27.3 ± 0.51	9%	0.0735
	THC	0.24 ± 0.018	0.26 ± 0.001	6%	0.4573
	THCVA	0.11 ± 0.004	0.12 ± 0.001	16%	0.0101
	THCV	<LOQ	<LOQ		

^z^ CBGA, cannabigerolic acid; CBG, cannabigerol; CBDA, cannabidiolic acid; CBD, cannabidiol; CBNA, cannabinolic acid; CBN, cannabinol; CBDVA, cannabidivarinic acid; CBDV, cannabidivarin; THCA, Δ^9^-tetrahydrocannabinolic acid; THC, Δ^9^-tetrahydrocannabinol; THCVA, tetrahydrocannabivarinic acid; THCV, tetrahydrocannabivarin; ^y^ *p*-values are according to Student’s *t*-test between photoperiod treatments; ^x^ these cannabinoids were below the limit of quantification (<LOQ); * *n* = 2 for this cultivar by photoperiod combination.

**Table 2:** Growth parameter responses (mean ± SE, *n* = 3) of *C. sativa* cultivars ‘Incredible Milk’ (IM) and ‘Gorilla Glue’ (GG) to 12 h and 13 h photoperiod treatments. Harvest index and total aboveground tissue data are based on dry weight (DW).

Cultivar	Parameter	12 h	13 h	Increase (%) ^z^	*p*-Value ^y^
IM	Growth index ^x^	807 ± 88.7	1460 ± 69.6	81	0.0045
	Harvest index ^w^	0.57 ± 0.001	0.59 ± 0.006	2	0.0844
	Total aboveground DW (g/plant)	128 ± 3.2	169 ± 5.3	32	0.0027
GG	Growth index	626 ± 121.2	972 ± 42.9	55	0.0543
	Harvest index	0.57 ± 0.008	0.60 ± 0.005	6	0.0279
	Total aboveground DW (g/plant)	117 ± 16.9	165 ± 2.5	41	0.0492

^z^ These values represent the percent increase in the respective parameter in the 13 h vs. 12 h photoperiod treatment; ^y^ *p*-values are according to Student’s *t*-test. ^x^ Growth index = [height × width_1_ × width_2_]/300 (Ruter, 1992) [10]. ^w^ Harvest index = total inflorescence FW/(total inflorescence FW + aboveground vegetative FW).

## Data Availability

All data from the study are included in the manuscript. Further inquiries can be directed to the corresponding author.

## Supplementary Materials

The following supporting information can be downloaded at: https://www.mdpi.com/article/10.3390/plants13030433/s1, Figure S1: Relative spectral photon flux distribution used in all experimental plots (Ahrens et al., 2023) [5]; Table S1: Temperature and relative humidity (mean ± SD) in each experimental plot for the duration of the trial at canopy-level height; Figure S2: Diagram of the experimental layout within a single growth chamber. The six individual compartments comprise three replications of each photoperiod treatment (12 h and 13 h) with three plants of each cultivar (Gorilla Glue (GG) and Incredible Milk (IM)); Figure S3: Diagram of one compartment bench setup showing placement of individual rockwool blocks with Incredible Milk (blue square) and Gorilla Glue (green square) plants, rockwool slabs (yellow rectangle), and the locations of individual drippers (red circle). Image is for illustrative purposes only; the individual elements are not to scale.
