# Supplementary material for: Longer Photoperiod Substantially Increases Indoor-Grown Cannabis’ Yield and Quality: A Study of Two High-THC Cultivars Grown under 12 h vs. 13 h Days
Source: Plants (Basel). 2024 Feb 1;13(3):433. doi: 10.3390/plants13030433 (PMC10857075; doi:10.3390/plants13030433)
Supplement: Supplementary file 1 [file plants-13-00433-s001.zip › plants-2838099-supplementary.pdf]

Supplementary Materials

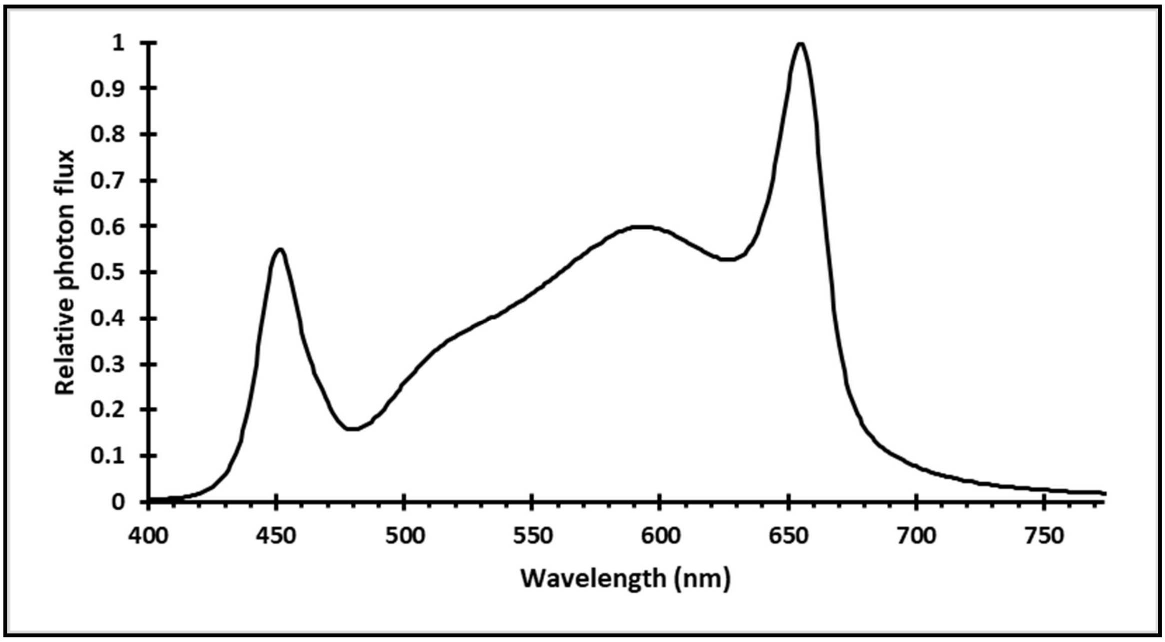

Figure S1. Relative spectral photon flux distribution used in all experimental plots (Ahrens et al., 2023) [5].

Table S1. Temperature and relative humidity (mean ± SD) in each experimental plot for the duration of the trial at canopy-level height.

**Photoperiod (Replication)**	**Lights**	**Temperature (°C)**	**Relative Humidity (%)**
13 h (1)	off	24.0 ± 0.73	66.7 ± 5.04
	on	25.3 ± 0.90	71.0 ± 4.80
12 h (1)	off	23.8 ± 0.97	68.6 ± 4.87
	on	25.9 ± 1.19	70.4 ± 5.25
13 h (2)	off	23.7 ± 0.74	69.6 ± 5.50
	on	24.7 ± 0.97	74.9 ± 4.95
12 h (2)	off	23.6 ± 0.84	70.2 ± 4.14
	on	24.8 ± 0.94	74.8 ± 4.74
13 h (3)	off	23.7 ± 0.70	69.2 ± 3.61
	on	25.6 ± 0.98	71.3 ± 4.67
12 h (3)	off	23.9 ± 0.74	67.2 ± 3.58
	on	25.1 ± 0.96	71.9 ± 4.99

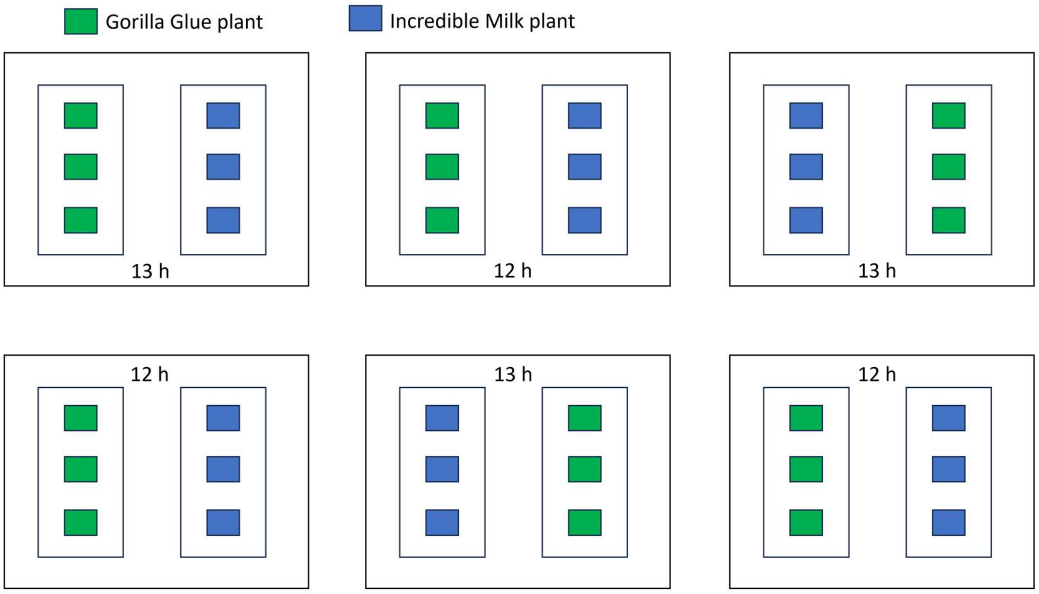

Figure S2. Diagram of the experimental layout within a single growth chamber. The six individual compartments comprise three replications of each photoperiod treatment (12 h and 13 h) with three plants of each cultivar (Gorilla Glue, "GG" and Incredible Milk, "IM").

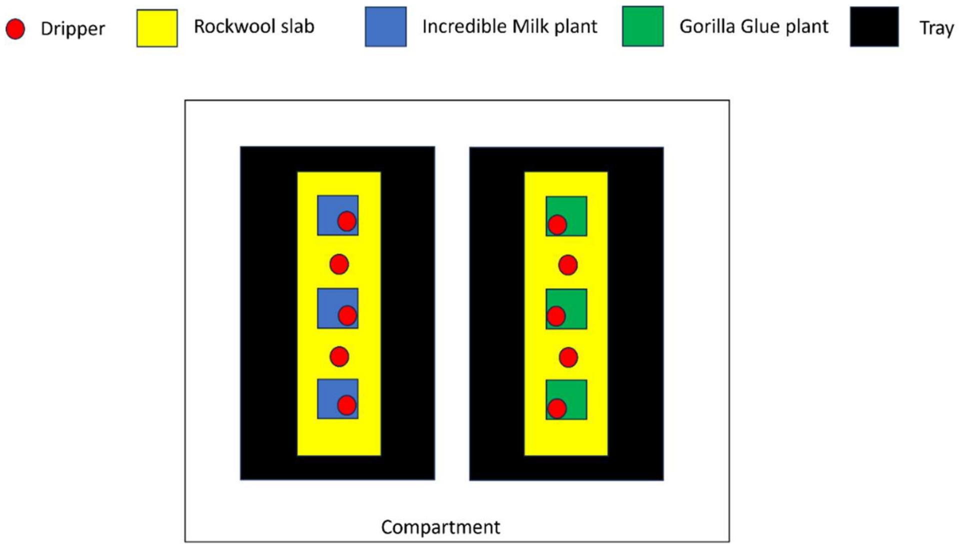

Figure S3. Diagram of one compartment bench setup showing placement of individual rockwool blocks with Incredible Milk (blue square) and Gorilla Glue (green square) plants, rockwool slabs (yellow rectangle) and the locations of individual drippers (red circle). Image is for illustrative purposes only; the individual elements are not to scale.
